# ZFP161 regulates replication fork stability and maintenance of genomic stability by recruiting the ATR/ATRIP complex

**DOI:** 10.1038/s41467-019-13321-z

**Published:** 2019-11-22

**Authors:** Wootae Kim, Fei Zhao, Rentian Wu, Sisi Qin, Somaira Nowsheen, Jinzhou Huang, Qin Zhou, Yuping Chen, Min Deng, Guijie Guo, Kuntian Luo, Zhenkun Lou, Jian Yuan

**Affiliations:** 1 Department of Molecular Pharmacology and Experimental Therapeutics, Mayo Clinic, Rochester, MN 55905 USA; 2 Department of Oncology, Mayo Clinic, Rochester, MN 55905 USA; 3 Mayo Clinic Medical Scientist Training Program, Mayo Clinic Alix School Of Medicine and Mayo Clinic Graduate School of Biomedical Sciences, Mayo Clinic, Rochester, MN 55905 USA; 4 Research Center for Translational Medicine, East Hospital, Tongji University School of Medicine, No. 150 Jimo Road, Shanghai, 200120 China; 5 Key Laboratory of Arrhythmia, Ministry of Education, East Hospital, Tongji University School of Medicine, No. 150 Jimo Road, Shanghai, 200120 China

**Keywords:** Cancer, Cancer, DNA replication, DNA replication

## Abstract

DNA replication stress-mediated activation of the ATR kinase pathway is important for maintaining genomic stability. In this study, we identified a zinc finger protein, ZFP161 that functions as a replication stress response factor in ATR activation. Mechanistically, ZFP161 acts as a scaffolding protein to facilitate the interaction between RPA and ATR/ATRIP. ZFP161 binds to RPA and ATR/ATRIP through distinct regions and stabilizes the RPA–ATR–ATRIP complex at stalled replication forks. This function of ZFP161 is important to the ATR signaling cascade and genome stability maintenance. In addition, ZFP161 knockout mice showed a defect in ATR activation and genomic instability. Furthermore, low expression of ZFP161 is associated with higher cancer risk and chromosomal instability. Overall, these findings suggest that ZFP161 coordinates ATR/Chk1 pathway activation and helps maintain genomic stability.

## Abstract (web summary)

The ATR pathway is active during DNA replication stress to maintain genome stability. Here the authors reveal the role of the zinc finger containing protein 161 (ZFP161) to facilitate replication fork stability by acting as a scaffold to facilitate the interaction between RPA and ATR/ATRIP.

## Introduction

DNA replication is an important biological process for genomic duplication. Faulty DNA replication leads to accumulation of mutations or chromosomal instability including deletion, insertion, and loss of heterozygosity, which contribute to the development of cancer. Spontaneous lesions in DNA replication are repaired by the DNA damage response (DDR) and repair system to maintain genomic stability^1,2^.

Errors in DNA replication increase stalled replication forks, which generate single strand DNA (ssDNA) and are toxic to cells. Stalled replication forks are sensed by replication protein A complex (RPA), a ssDNA-binding protein^3–5^. The RPA–ssDNA complex recruits the DDR protein ATR interacting protein (ATRIP), which subsequently recruits ATR to DNA damage sites^6,7^. The RPA-ssDNA complex also act as a platform for recruiting the Rad17-Replication factor C (RFC) complexes to DNA damage sites, which in turn load the 9-1-1 complex and TopBP1^8–10^. ATR is activated by a direct interaction with TopBP1^11,12^. ATR can also be activated by ETAA1, which is independent of TopBP1^13,14^. Activated ATR further phosphorylates and activates Chk1, which stabilizes and restarts stalled replication forks through various downstream effectors^6,9,15–17^.

ZFP161, also called ZBTB14, is a zinc finger protein and belongs to the Krüppel type ZFP family. Krüppel type ZFP family factors regulate a diverse cellular functions such as transcription, metabolism, differentiation, apoptosis and tumorigenesis^18^. ZFP161 contains four Zinc finger domains and one BTB/POZ domain^19^. It is known to bind to GC-rich site-specific DNA sequence and act as a transcription regulator. ZFP161 acts as a transcriptional activator of dopamine transporter (DAT), interleukin 6 (IL-6), leukemia inhibitory factor (LIF) and a transcriptional repressor of FMR1 by binding to promoters of these genes. ZF5, which is mouse gene homolog, has been shown to be a repressor of c-Myc and thymidine kinase^20–24^. In Xenopus, Zbtb14 is important for neural development as it promotes Wnt signaling^25^. Furthermore, the ZFP161 gene in human is located at 18p11.21-pter, which might be associated with Holoprosencephaly 4, a genetic disease that causes brain malformation^26^. However, little is known about the function of ZFP161 in DNA damage response and repair. We show that ZFP161 is involved in replication related DDR and localizes to stalled forks via interaction with RPA complex. ZFP161 also interacts with ATR/ATRIP and is important for ATR/ATRIP recruitment and full activation of ATR-Chk1 signaling to correct replication errors. In addition, we show defects in ATR activation and subsequent genomic instability in ZFP161 knockout mice. Furthermore, low expression of ZFP161 is associated with higher cancer risk and chromosomal instability. Overall, we show that ZFP161 is an important regulator of replication stress response by facilitating the complex of RPA and ATR/ATRIP.

## Results

### ZFP161 participates in the activation of ATR signaling pathway

**Fig. 1:**
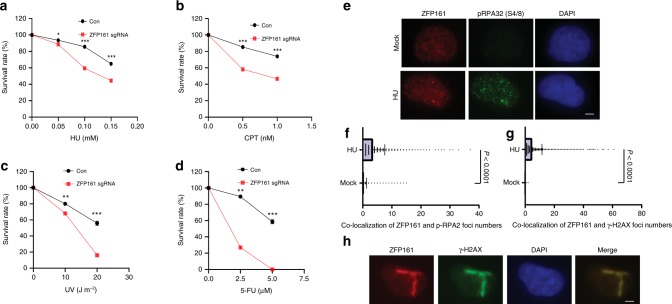
ZFP161 is involved in the replication stress response. **a**–**d** HCT116 cells were plated and treated with Hydroxyurea (HU, mM) (**a**), Camptothecin (CPT, nM) (**b**), Ultraviolet (UV, J m^−2^) (**c**), and Fluorouracil (5-FU, µM) (**d**). After 14 days, colony numbers were counted. The graphs represent mean ± S.D., two-tailed, paired *t*-test. **e** The co-localization of phospho-RPA32 (S4/S8) and ZFP161 was determined by immunofluorescence. **f**, **g** Quantification for co-localization of ZFP161 per cell with phospho-RPA32 (S4/S8) (**f**), and γH2AX (**g**). The graphs represent mean ± S.D., two-tailed, unpaired *t*-test. **h** Cells were stained with ZFP161 and γH2AX 2 h after microirradiation. Scale bars, 10 µm. Source data are provided as a Source Data file.

Proteins possessing zinc finger domain potentially bind to DNA, which may be involved in various cellular processes such as transcription regulation, DNA modification, and DNA damage repair. Multiple zinc finger proteins are reported to be involved in DNA damage response such as BRCA1, PARP1, and ZMYM3^27^. We are interested in identifying zinc finger proteins that have not been previously studied in the DNA damage response. Here we explored the role of a zinc finger protein, ZFP161, in DNA damage response. First, we generated ZFP161 knockout cell lines using CRISPR/Cas9 system and tested whether ZFP161 deficiency sensitizes cells to DNA damaging agents. The loss of ZFP161 caused hypersensitivity to hydroxyurea (HU), camptothecin (CPT), ultraviolet (UV) irradiation, and Fluorouracil (5-FU), but not ionizing radiation (IR), cisplatin or olaparib (Fig. 1a–d, Supplementary Fig. 1A–E). ZFP161 knockout clones were validated by sequencing. Sequencing results revealed that both clone #1 and #2 codon were frameshifted (Supplementary Fig. 1F). These data suggest that ZFP161 may play a role in replication stress response. To further confirm the role of ZFP161 in DDR, we examined its cellular localization following DNA damage. As shown in Fig. 1e–h, ZFP161 co-localized with phospho-RPA32 (S4/8) and γ-H2AX (a marker of DNA damage) following hydroxyurea (HU) or UV microirradiation treatment, suggesting that ZFP161 might be involved in replication related DNA damage response.

### ZFP161 is involved in ATR related DNA damage response

**Fig. 2:**
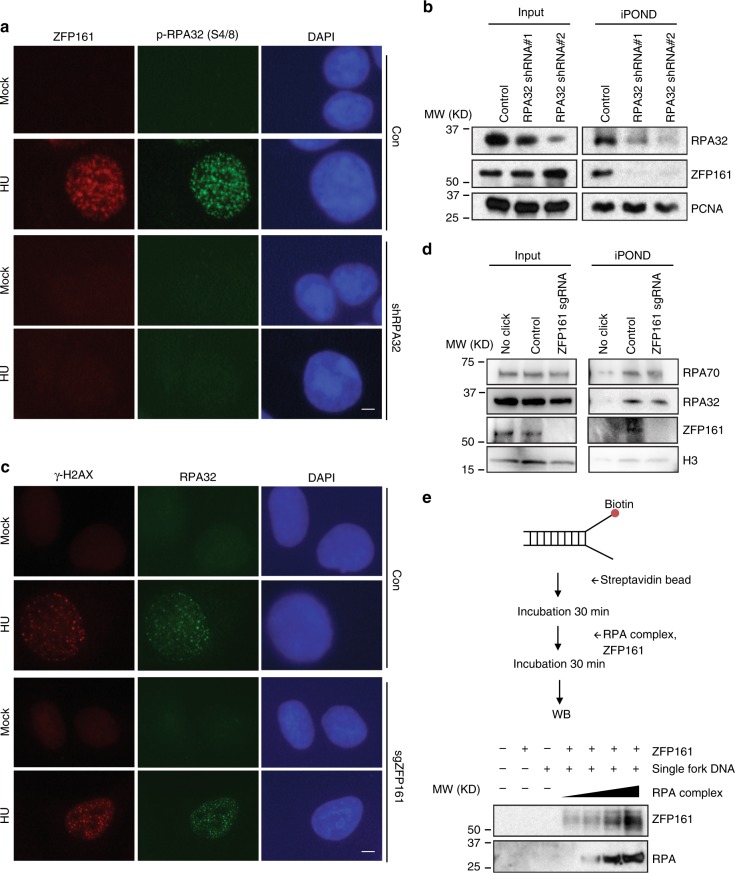
ZFP161 is enriched at replication forks. **a** Cells were depleted of RPA32 using shRNA. The co-localization of phospho-RPA32 (S4/S8) and ZFP161 were determined by immunofluorescence. **b** RPA32 deficient HCT116 cells were incubated with EdU and HU. Replication fork proteins were isolated by iPOND and immunoblotted with indicated antibodies. **c** Cells were depleted of ZFP161 using sgRNA. The co-localization of RPA32 and γH2AX were determined by immunofluorescence. **d** ZFP161 deficient HCT116 cells were incubated with EdU and HU. Replication fork proteins were isolated by iPOND and immunoblotted with indicated antibodies. **e** Purified GST-ZFP161 protein and RPA complex were incubated with biotin labeled single fork DNA. The proteins retained on DNA were determined by immunoblotting. Scale bars, 10 µm. Source data are provided as a Source Data file.

Next, we studied how ZFP161 is recruited to DNA damage sites. RPA32 is a sensor of ssDNA and helps recruit many downstream factors involved in the replication stress response. Interestingly, knocking down RPA32 abolished ZFP161 foci formation, suggesting that ZFP161 is recruited to single strand breaks (SSBs) in an RPA-dependent manner (Fig. 2a). Because of RPA’s role in replication stress, we next asked whether ZFP161 is recruited to replication forks. To assess this, we performed iPOND (isolation of proteins on nascent DNA) assays. Indeed, ZFP161 localized to replication forks. In RPA-deficient cells, ZFP161 failed to accumulate at replication forks (Fig. 2b). However, ZFP161 did not affected RPA foci formation or accumulation at replication forks (Fig. 2c, d), suggesting that ZFP161 is downstream of RPA complex. These results were validated in vitro as well. We performed in vitro DNA binding assays using purified RPA complex and GST-ZFP161. ZFP161 did not bind strongly with DNA, and RPA enhanced ZFP161’s binding to single stranded DNA fork (Fig. 2e). Taken together, these results suggest that ZFP161 is recruited to replication forks by the RPA complex.

**Fig. 3:**
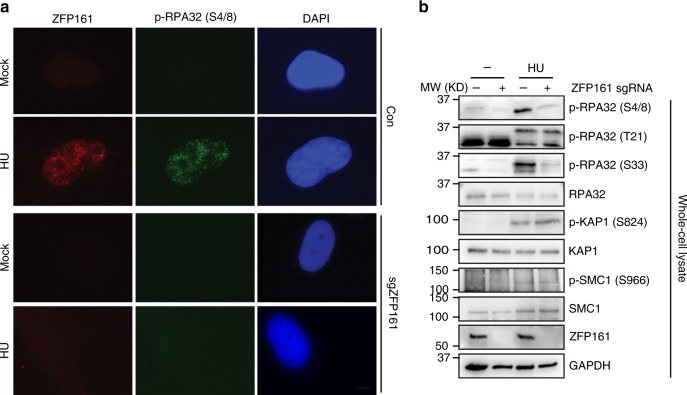
ZFP161 is important for ATR activation. **a** The co-localization of phospho-RPA32 (S4/S8) and ZFP161 was determined by immunofluorescence in control or ZFP161-deficent cells. **b** After 10 mM HU treatment for 2 h, phosphorylation of DNA damage related proteins was determined by immunoblotting in ZFP161-knockout HCT116 cells. Scale bars, 10 µm. Source data are provided as a Source Data file.

Following replication stress, RPA directly binds to ssDNA and recruits ATRIP/ATR complex through direct interactions with ATRIP^6^. Activated ATR triggers cell cycle checkpoint and DNA damage repair through phosphorylating CHK1, RPA and other factors. To examine the role of ZFP161 in the replication stress response, we examined the activation of ATR signaling cascades. As shown in Fig. 3a, b and Supplementary Fig. 1G–H, ZFP161 deficiency diminished ATR-dependent RPA32 phosphorylation (S4/S8 and S33) and also significantly decreased Chk1 phosphorylation following HU, UV, and CPT treatment. To confirm these results, cells were treated with different inhibitors of the DNA damage response pathway (DNA-PK, ATM and ATR) and RPA32 phosphorylation was determined. As shown in Supplementary Fig. 1I, only treatment with ATR inhibitor diminished RPA32 S4/8 and S33 phosphorylation upon co-treatment with 10 mM HU for 2 h. These results are consistent with previous reports^28,29^ and suggest that ZFP161 is important for full activation of ATR signaling cascades. Interestingly, we found only ATR-dependent RPA32 phosphorylation was decreased in ZFP161 deficient cells when compared to ZFP161 proficient cells. Knocking out ZFP161 did not affect ATM phosphorylation and ATM-dependent SMC1, KAP1, and Chk2 phosphorylation after ionizing radiation (IR) (Supplementary Fig. 1J–K), suggesting a specific role of ZFP161 in ATR activation. In order to study how ZFP161 regulates ATR activation, we examined the expression of ATR or other factors involved in ATR activation, such as the RPA complex, Rad17, and TopBP1. However, the expression of these factors was not affected in ZFP161-deficient cells (Supplementary Fig. 1H). Taken together, our data suggest that ZFP161 is involved in the replication stress response pathway and promotes ATR activation.

### ZFP161 is a mediator protein of the RPA-ATRIP axis

**Fig. 4:**
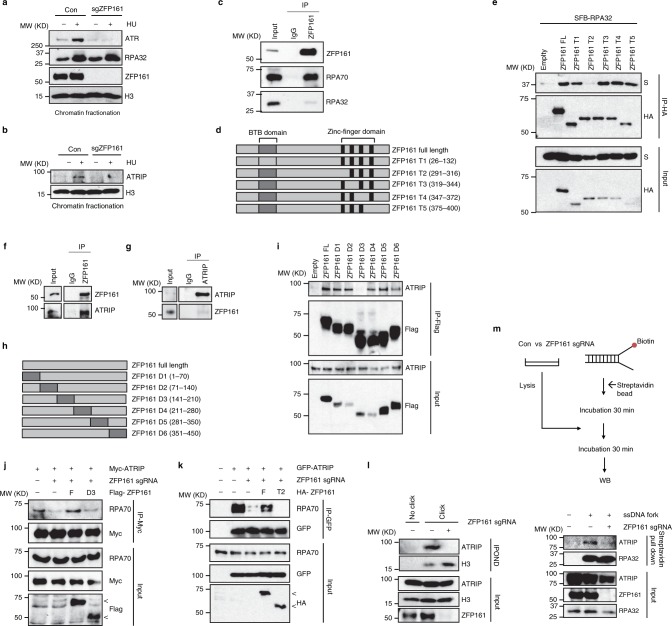
ZFP161 mediates the RPA-ATRIP interaction. **a**, **b** Chromatin fractionations from HCT116 cells in presence or absence of ZFP161 following 10 mM HU treatment for 2 h were used for immunoblotting with indicated antibodies. **c** Whole cell lysates from HCT116 cells were used for immunoprecipitation with ZFP161 antibody. **d** The schema of ZFP161 constructs used to identify the minimal region required for its interaction with RPA32. **e** HEK293T cells were transfected with S-tagged RPA32 and ZFP161 vectors. After 48 h, the interaction between ZFP161 and RPA32 was determined by immunoprecipitation and immunoblotting with indicated antibodies. **f**, **g** Whole cell lysates from HCT116 cells were used for immunoprecipitation with ZFP161 (**f**), or ATRIP (**g**) antibodies. **h** The schema of ZFP161 constructs used to identify the minimal region required for its interaction with ATRIP. **i** HEK293T cells were transfected with the indicated ZFP161 vectors. Aftter 48 h, ZFP161 and ATRIP interaction was determined by immunoprecipitation and immunoblotting with indicated antibodies. **j**, **k** HCT116 cells expressing or lacking ZFP161 were transfected with constructs encoding Myc-ATRIP, ZFP161 (WT) and D3 mutant (**j**) or T2 mutant (**k**). Whole cell lysates were immunoprecipitated with Myc antibody and immunoblotted with indicated antibodies. **l** ZFP161 deficient HCT116 cells were incubated with EdU and HU. Replication fork proteins were isolated by iPOND and immunoblotted with indicated antibodies. **m** Biotin labeled single fork DNA was incubated with HCT116 whole cell lysates and pulled down with streptavidin beads. The interaction of ATRIP and RPA on DNA was determined by immunoblotting. < : ZPF161 full length and mutants bands. Source data are provided as a Source Data file.

When we explored whether ZFP161 affects the accumulation of DDR factors on chromatin following replication stress, we found that the chromatin association of both ATR and ATRIP were significantly decreased in ZFP161-deficient cells, while RPA accumulation was not affected (Fig. 4a, b). The results above led us to hypothesize that ZFP161 is a mediator protein in the RPA-ATRIP axis. We found an interaction between ZFP161 and RPA in unstressed cells (Fig. 4c). Moreover, the ZFP161-RPA interaction did not change following DNA damage (Supplementary Fig. 2A). To map the interaction between ZFP161 and RPA, we generated and purified recombinant GST-tagged ZFP161 (WT, N-, M-, C-terminal fragments) and RPA complex in bacterial expression system. GST-ZFP161 was able to pull down RPA from cell lysates and directly interact with RPA70 and RPA32 in cell-free systems (Supplementary Fig. 2B - D). In addition, the C-terminus of ZFP161 that contains several zinc-finger domains was sufficient to bind RPA (Supplementary Fig. 2C). To further narrow down the region of ZFP161 that interacts with the RPA complex, we generated zinc finger domain truncated mutants of ZFP161 and performed immunoprecipitation assays. We found that the ZFP161 T2 (amino acid 291–316), which contains the first zinc finger domain in ZFP161, is required to interact with RPA32 (Fig. 4d, e). We further hypothesized that ZFP161 interacts with ATRIP and is involved in the recruitment of ATRIP to chromatin. Indeed, ZFP161 interacted with ATRIP as suggested by the co-immunoprecipitation (Co-IP) experiments using anti-ZFP161 antibody (Fig. 4f). Reciprocal Co-IP with anti-ATRIP antibody also showed that ATRIP interacts withZFP161 (Fig. 4g). We also confirmed the ATRIP and ZFP161 interaction using ectopic IP (Supplementary Fig. 2E). These interactions were unaffected by replication stress (Supplementary Fig. 2F). Next, we performed GST pulldown assays using GST-ZFP161 fragments to identify the domain(s) of ZFP161 responsible for ATRIP interaction. GST-ZFP161 was able to interact with ATRIP through the M-terminal fragments (M) of ZFP161 (Supplementary Fig. 2G). We further narrowed down the region required for ATRIP binding and found that deletion of amino acid 141–210 of ZFP161 (D3) abrogated the interaction between ZFP161 and ATRIP (Fig. 4h, i).

We hypothesized that, if ZFP161 functions as a mediator factor between ATRIP and RPA complex, the RPA-ATRIP interaction should be compromised in the absence of ZFP161. Indeed, we found that this interaction was significantly decreased in ZFP161 deficient cells (Fig. 4j, k). More importantly, this interaction was restored by the ectopic expression of wild type (WT) ZFP161 but not the D3 or T2 mutant which disrupt the interaction with ATRIP and RPA, respectively (Fig. 4j, k). To further test whether ZFP161 helps to recruit ATRIP to replication forks, we performed iPOND assays using ZFP161 knockout cells. We found that ATRIP failed to be recruited to stalled replication forks in ZFP161-deficient cells (Fig. 4l). Furthermore, we generated biotin labeled DNA mimicking replication fork and checked ATRIP recruitment using ZFP161-proficient and -deficient cells. ATRIP was recruited to replication forks in ZFP161 proficient cells but not in ZFP161-deficient cells (Fig. 4m). These results suggest that ZFP161 is recruited by RPA and is involved in ATRIP recruitment. Taken together, our data suggest that ZFP161 is a key adaptor protein in the replication stress response pathway and mediates the interaction between RPA and ATRIP.

### ZFP161 is required for stalled replication fork restart

**Fig. 5:**
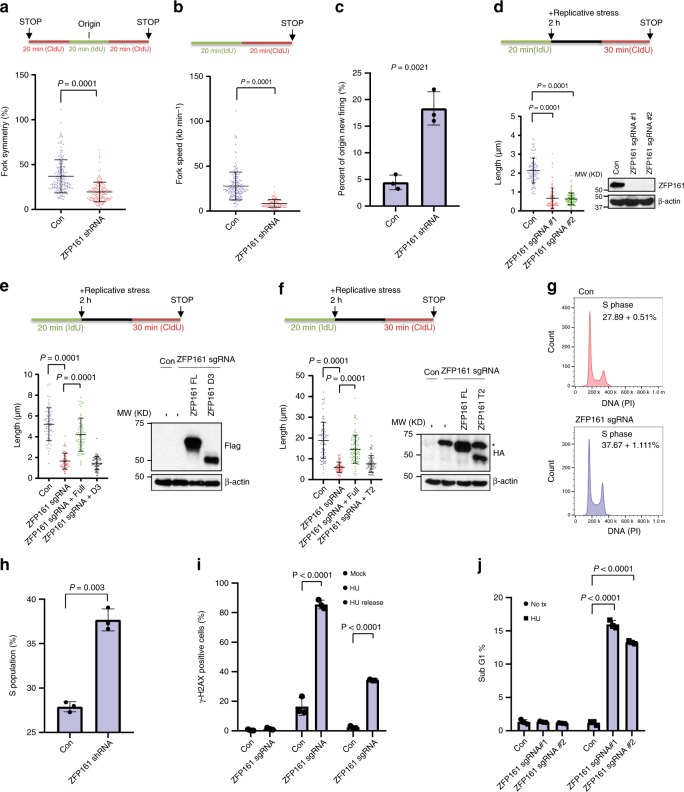
ZFP161 promotes stalled replication fork restart **a**, **b** HCT116 cells were labeled with IdU and CldU for indicated time. Fork symmetry **a** and fork speed **b** were determined by measuring the length of CIdU (red) track. **c** The percentage of new origins (red only fibers) was quantified. *n* = 313 fibers for WT and *n* = 285 fibers for ZFP161 knockout. **d**–**f** Cells were labeled with IdU for 20 min and then cells were incubated with 4 mM HU for 2 h. After incubation, cells were labeled with CldU for 30 min. ZFP161 KO cells (**d**) or KO cells reconstituted with full length ZFP161 and the D3 mutant (**e**), or the T2 mutant (**f**) were used. DNA fibers were stretched on a microscope slide, stained with IdU and CldU antibodies, imaged, and the lengths of fiber tracks measured. The graphs represent mean ± S.D., two-tailed, unpaired *t*-test. **g**, **h** Cell cycle distribution was determined by flow cytometry in cells either lacking or expressing ZFP161 (**g**). S phase populations were analyzed by Modifit (**h**). **i**, **j** HCT116 cells were treated with 2 mM HU for 24 h and released. Cells were collected and γ-H2AX (**i**) and sub G1 population (an indicator of cell death) (**j**) were determined by flow cytometry. *: unspecific bands The graphs represent mean ± S.D., two-tailed, paired *t*-test; *n* = 3 independent experiments. Source data are provided as a Source Data file.

The RPA complex and ATR signaling pathway are critical for DNA replication and recovery of stalled replication. To investigate whether ZFP161-deficient cells have DNA replication defects under replication stress, we performed DNA fiber assays. In the absence of replication stress, ZFP161-deficient cells showed asymmetrical and slower replication than ZFP161 proficient cells (Fig. 5a, b, Supplementary Fig. 3A). Interestingly, we found an increase in origin firing in ZFP161 deficient cells (Fig. 5c, Supplementary Fig. 3A). Under replication stress, ZFP161-deficient cells exhibited significant reduction in replication strand lengths when compared to control cells (Fig. 5d, Supplementary Fig. 3A). Additionally, this phenomenon was complemented by the expression of wild type ZFP161, but not the D3 or T2 mutant (Fig. 5e, f, Supplementary Fig. 3A). These results suggest that ZFP161 and its interaction with RPA and ATRIP is important for replication fork stability.

### Loss of ZFP161 sensitizes cells to DNA damage

The unstressed ZFP161-deficient cells showed slight increase in S phase population compared to ZFP161 proficient cells (Fig. 5g, h). After HU treatment to induce replication stress, ZFP161 deficient cells demonstrated a slower recovery from the insult compared to ZFP161 proficient cells (Supplementary Fig. 3B). Since ZFP161 is important for ATR activation and replication fork stability, we hypothesized that ZFP161 knockout cells are more sensitive to replication stress. To test this hypothesis, we examined γ-H2AX signals (Fig. 5i, Supplementary Fig. 3C) and cell death (Fig. 5j, Supplementary Fig. 3D) after HU treatment using ZFP161 deficient cells. As shown in Fig. 5g–j, ZFP161-deficient cells accumulated more cellular DNA damage and had decreased viability. Taken together, our results indicate that ZFP161 mediates the RPA-ATR signaling pathway and is important for replication fork protection, stalled replication restart and physiologic processes.

### ZFP161 maintains genomic stability in vivo

**Fig. 6:**
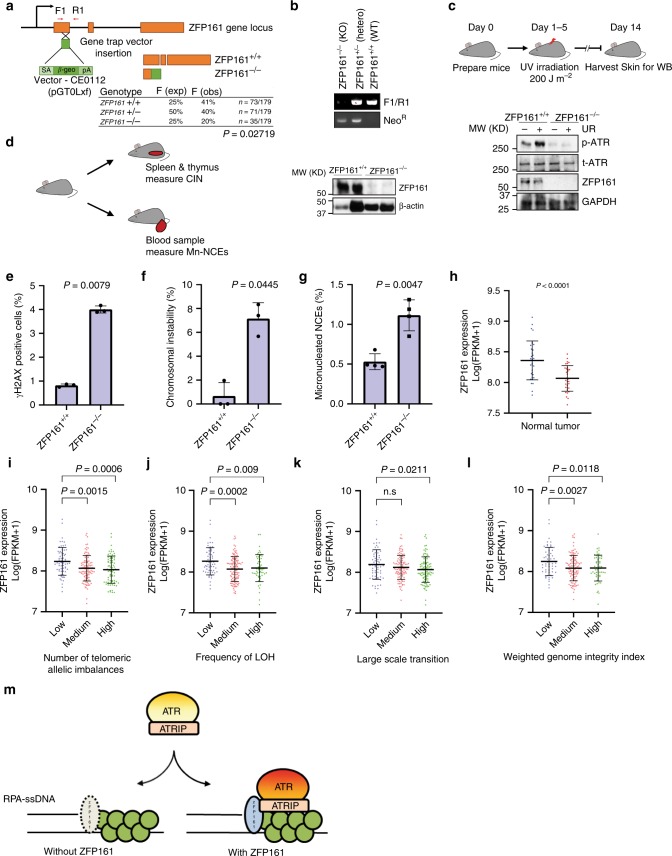
ZFP161 Maintains Genomic Stability. **a** Generation and characterization of ZFP161 KO mice. A. Schematic representation of wild- type and gene-trapped alleles in the ZFP161 genomic locus. **b** PCR and Western blot analysis of genomic DNAs and lysates from ZFP161^+/+^, ZFP161^+/−^, and ZFP161^−/−^ mice. F (exp) and F (obs) are expected and observed Mendelian frequencies, respectively, and n, the number of pups genotyped at three weeks after birth. Two-sided Fisher’s exact test, *p* = 0.02719. **c** Mice were treated with UV and ATR signaling was determined by Western blot of skin tissues. **d**–**g** Genomic stability was examined by measuring γH2AX (**e**), metaphase spread (**f**), and micronucleated normochromic erythrocytes (Mn-NCE, CD71-PI + ) (**g**). *n* = 3 independent experiments. 3 mice per genotypes, 50 metaphases per mice. **h**–**l** TCGA database analysis. Tumor mRNA expression levels were obtained from TCGA for 32 paired patient samples (**h**). Correlation of ZFP161 expression levels with chromosomal instability levels. Number of telomeric allelic imbalances (**i**), frequency of LOH (**j**), large scale transition (**k**), and weighted genome integrity index (**l**). **m** Diagram showing that ZFP161 mediates the recruitment of ATRIP/ATR to repair stalled replication fork. The graphs represent mean ± S.D., two-tailed, paired *t*-test. Source data are provided as a Source Data file.

Accumulated replication stress commonly leads to higher levels of DNA damage and genomic instability. To investigate whether ZFP161 deficiency induces genomic instability in vivo, we generated ZFP161 knockout mice. We found that the number of the ZFP161 knockout pups was less than estimated by Mendelian proportions (Fig. 6a, b). We first examined ATR pathway signaling upon UV irradiation. As shown in Fig. 6c, ATR activation was compromised in ZFP161 deficient mice. We also examined spontaneous genomic instability using several methods in ZFP161-deficient mice (Fig. 6d). ZFP161^−/−^ splenocytes showed significant increase in the proportion of γ-H2AX positive cells compared to wild-type splenocytes (Fig. 6e, Supplementary Fig. 4A). We also used metaphase spreads of activated T cells to examine genomic stability. As shown in Fig. 6f and Supplementary Fig. 4B, spontaneous chromosomal instability was significantly increased in ZFP161^−/−^ T cells. However, ZFP161 deficiency does not affect T cell cellularity and T cell differentiation (Supplementary Fig. 5). We also examined hematopoietic cells for evidence of chromosomal instability. Micronuclei are formed from chromosomal instability and can be easily detected in normochromic erythrocytes (NCEs). ZFP161^−/−^ NCEs showed significantly increased micronuclei compared to wild-type cells (Fig. 6g, Supplementary Fig. 4C). Overall, our results suggest that ZFP161 plays a critical role in maintenance of genomic stability.

### ZFP161 level negatively correlates with genomic instability

The above data suggest that ZFP161 deficiency leads to compromised replication stress response and increased genomic instability. Because genomic instability is a hallmark and driver of tumorigenesis, we next explored a potential role of ZFP161 in tumor. By analyzing the colorectal TCGA database, we found that ZFP161 transcript levels are significantly lower in colon adenocarcinoma relative to normal tissues (Fig. 6h). Furthermore, high chromatin instability represented by telomeric allelic imbalances, loss of heterozygous (LOH), large scale transition, and weighted genome integrity index significantly correlated with low expression ZFP161 in colon adenocarcinoma (Fig. 6i–l). Taken together, our results suggest that expression of ZFP161 is inversely related to tumorigenesis and chromosomal instability, consistent with a role of ZFP161 in the maintenance of genomic stability.

## Discussion

Apprehending how the ATR/ATRIP complex is recruited and regulated at stalled replication forks or ssDNA damage sites are critical for understanding the ATR signaling pathway. In replication stress response pathway, ATR is responsible for maintaining replication related genomic stability. The RPA complex is a key player in initiating DNA-damage checkpoint signaling, replication, and stalled replication fork restart by binding with ssDNA. The ssDNA-RPA binding not only protects ssDNA from nuclease activity, but also forms a platform for the recruitment of proteins (ATR/ATRIP, Rad17, 9-1-1, and TopBP1), which is required for ATR recruitment and activation^6,16^.

Here, we identified ZFP161 as a key player that is involved in RPA dependent ATR activation for repairing of stalled replication forks. Our study shows that ZFP161 is recruited by the RPA complex to ssDNA and then stabilizes RPA-ATR/ATRIP interaction for ATR signaling. Previously, ATR/ATRIP was proposed to directly interact with RPA complex in vivo and in vitro^6^. We found that ZFP161 could further stabilize this interaction. In the absence of ZFP161, ATR/ATRIP recruitment to replication fork is significantly decreased. Although ZFP161 is implicated in transcription regulation, we did not detect any effect on the expression of key factors in the ATR pathway. Instead, we found that ZFP161 interacts with RPA and ATRIP through distinct regions and enhances the RPA-ATRIP interaction. Taken together, our results demonstrate that ZFP161 interacts with RPA and helps build a stable platform for recruiting ATR/ATRIP.

Furthermore, ZFP161 knockout mice displayed a phenotype of impaired ATR signaling and replication. Previous studies showed that depletion of ATR signaling pathway proteins (ATR, ATRIP, Rad17, etc.) leads to chromosome breaks and embryonic lethality^30^. Although ZFP161 KO mice are viable, the number of the ZFP161 knockout pups was less than estimated by Mendelian proportions. Depletion of ZFP161 impairs ATR signaling but does not totally block ATR activation. This might explain why ZFP161 KO mice are viable while ATR KO mice are embryonically lethal. ZFP161 KO also results in spontaneous genomic instability in vivo. In addition, by analyzing the genomic stability signature in colorectal cancer, we uncovered an association between low expression of ZFP161 and genomic instability. Collectively, our study provides evidence of the relationship between ZFP161 and genomic instability. It suggests that ZFP161 is a missing piece of replication stress response cascade for ATR activation.

In summary, our study clarifies ZFP161 as a regulator of ATR signaling. ZFP161 functions as an adaptor protein for connecting ssDNA-RPA and ATRIP/ATR complex, which activates ATR signaling pathway under replication stress and maintains genomic stability (Fig. 6m).

## Methods

### Cell culture and inhibitor

HEK293T (CRL-3216), HCT116 (CCL-247) and U2OS (HTB-96) cell lines were purchased from ATCC and cultured in Dulbecco’s Modified Eagle’s Medium or McCoy’s 5A supplemented with 10% fetal bovine serum (FBS) at 37 °C in 5% (v/v) CO_2_.The following inhibitors were used: VE-822 (Selleckchem: s7102), NU7441 (selleckchem:S2638), and KU55933 (abcam: ab120637).

### Plasmids and antibodies

pCMV6–Myc-ZFP161 vector was purchased from ORIGENE. ZFP161 was sub-cloned into pGEX4T-2 (Clontech), pCMV, and pLVX3 vectors. All ZFP161 truncated mutants were generated by site-directed mutagenesis. Rabbit anti-ZFP161 antibodies were generated by immunizing rabbits with GST-ZFP161-M (amino acids 150-300) or GST-ZFP161-C (amino acids 349-450). Anti-phospho KAP1 (S824) antibody was generated by Cocalico biologicals, anti-phospho ATR (T1989) and anti-phospho RPA32 (S4/8) antibodies were generated by GenScript. The following antibodies were used: anti-phospho RPA32 (S4/8) (Bethyl Laboratories: A300-245A, 1:2000), anti-phospho RPA32 (T21) (Abcam: ab109394, 1:2000), anti-phospho RPA32 (S33) (Bethyl Laboratories: A300-246A-M, 1:2000), anti-RPA70 (Bethyl Laboratories: A300-241A, 1:2000, 1: 5000), anti-RPA32 (Bethyl Laboratories: A300-244A, 1:5000, Santa Cruz Biotechnology: sc-56770, 1:2000), anti-γH2AX (EMD Millipore Crop: 2884537, 1:1000, Bethyl Laboratories: A300-081A, 1:1000), anti-PCNA (CST: 2586, 1: 2000), anti-H3 (Proteintech, 17168-1-AP, 1:5000), anti-phospho Chk1 (S345) (CST: 2348, 1:1000), anti-Chk1 (Santa Cruz Biotechnology: sc-8408, 1:2000), anti-phospho Chk2 (T68) (CST: 2197S, 1:1000), anti-Chk2 (CST: 6334, 1:2000), anti-phospho ATR (T1989) (Genetex: GTX128145, 1:1000), anti-ATR (CST: 13934, 1: 1000, Abcam: ab2905, 1:1000, Novus: nb100-308, 1:1000), anti-TopBP1 (Bethyl Laboratories: A300-111A, 1:1000), anti-Rad17 (Santa Cruz: sc-17761, 1:1000), anti-ATRIP (CST: 2737, 1:1000), anti-GAPDH (Proteintech: 60004-1-lg, 1:10,000), anti-β-actin (Sigma: A2228, 1:2000), anti-HA (Santa Cruz: sc805, 1:2000, Sigma: H6908, 1:2000), anti-Flag (Sigma: F1804, 1:2000, F7425, 1: 2000), anti-Myc (Santa Cruz: sc-40, 1: 1000, sc764, 1:1000), anti-GFP (Santa Cruz: sc-9996, 1:1000), anti-CD71 (eBioscience: 11-0711-82, 1:100), anti SMC1 (Epitomics: 2437-1, 1:1000), anti-phospho SMC1 (S957) (CST: 4805, 1:1000), anti-phospho SMC1 (S966) (Bethyl Laboratories: A300-050A, 1:1000), and anti-KAP1 (Bethyl Laboratories: A300-274A, 1:1000).

### shRNAs and sgRNAs

All ZFP161 shRNAs were purchased from Sigma. ZFP161 shRNA #1: TTGATAGTTCTTCGGTCATAG, ZFP161 shRNA #2: GACATGAAGTTTGAGTATTTG. ZFP161 knockout HCT116 and U2OS cells were generated using CRISPR. Briefly, two ZFP161 sgRNAs, CACCGGGGAAGACGTTTTCTGATGA and CACCGGGCAGGCAATCTGCTCCCGA, were inserted into LentiCRISPR v2 (Addgene). These shRNA and sgRNA vectors were used for lentiviral infection.

### DNA transfection, virus packaging and lentiviral infection

All DNA transfections were performed using TransIT-X2 (MIRUS Bio). Lentiviruses for infection of HCT116 and U2OS cells were packaged in HEK293T cells using targeted shRNA, sgRNA, pMD2.G and pSPAX2. Media containing lentivirus was collected forty-eight hours after transfection. Harvested medium was added to the cells for further experiments with 8 µg ml^−1^ polybrene for enhanced infection efficiency.

### Immunofluorescence

U2OS cells were cultured on coverslips 24 h before experiments. Cells were fixed with methanol: acetone (1:1) at −20 °C for 20 min. Cells were washed two times using PBS. Before staining, cells were blocked with 5% goat serum for 30 min. The primary antibodies were diluted in PBS containing 1% BSA and incubated with cell for 1 h for room temperature. After washing, secondary antibodies were diluted in PBS containing 1% BSA and incubated with cell at room temperature for 1 h. Cells were washed two times and incubated with DAPI at room temperature for 5 min. After washing, cells were mounted with anti-fade solution and visualized using a Nikon eclipse 80i Fluorescence microscope.

### Laser micro-irradiation

For laser micro-irradiation, U2OS cells were cultured on glass-bottom dish (MatTek Corporation). Laser micro-irradiation was performed using a Micropoint Laser Illumination and Ablation system (ANDOR). High energy UV laser generated DNA breaks. After 30 min, cells were fixed and stained with indicated antibodies.

### Immunoprecipitation and immunoblotting

For immunoprecipitation, cells were lysed with high salt NETN buffer (20 mM Tris–HCl, pH 8.0, 500 mM NaCl, 1 mM EDTA, and 0.5% NP-40) for 10 min and diluted with NET buffer (20 mM Tris–HCl (pH 8.0), 1 mM EDTA, and 0.5% NP-40). Whole cell lysates were centrifuged at 12,000 rpm for 10 min. Cell lysates were incubated with antibody and protein A beads (Amersham Biosciences) or GST-ZFP161 beads at 4 °C for 2 h or overnight. After incubation, beads were washed three times with NETN buffer. The bound proteins on the beads were eluted with 2× Laemmli buffer and loaded on SDS-PAGE and immunoblotted with the indicated antibodies. For immunoblotting, cells were lysed in NETN buffer (20 mM Tris-HCl, pH 8.0, 100 mM NaCl, 1 mM EDTA, and 0.5% NP-40) for 10 min. Whole cell lysates were centrifuged at 12,000 rpm for 10 min. Supernatants were heated at 95 °C for 10 min in 2× Laemmli buffer pellets were lysed using 0.2 N HCl for 20 min at 4 °C then centrifuged at 12,000 rpm for 10 min. After centrifugation, supernatants were neutralized with 0.2 M NaOH. Samples were heated at 95 °C for 10 mins in 2× Laemmli buffer and loaded to SDS-PAGE and immunoblotting with the indicated antibodies. Western blots were analyzed by Tanon 5200 imaging system or Kodak X-OMAT-2000A. All of uncropped blots are available in source data file.

### iPOND (isolation of proteins on nascent DNA)

iPOND assay was performed according to protocol^31^. Briefly, cells were labeled with 10 µM EdU for 20 min and then washed with washing buffer (0.5% bovine serum albumin in PBS). After washing, cells were incubated in media with 4 mM HU for 2 h. Cells were fixed with 1% formaldehyde in PBS for 20 min at room temperature. Next, formaldehyde was quenched with 1.25 M glycine. Cells were harvested, permeabilized with 0.25% Triton in PBS for 30 min, and incubated with click reaction buffer (1 mM biotin azide, 100 mM CuSO_4_, 20 mg ml^-1^ sodium L-ascorbate in PBS). After click reaction, cells were sonicated with lysis buffer (1% SDS in 50 mM Tris-HCl, pH 8.0) and centrifuged at 14,000 rpm for 10 min. Biotin labeled lysates were incubated overnight at 4 °C with streptavidin beads. After incubation, beads were rinsed twice with cold lysis buffer for 5 min each, once with 1 M NaCl, and twice with cold lysis buffer. Beads were heated at 95 °C in 2× Laemmli buffer for 30 min, loaded onto SDS-PAGE and immunoblotted with the indicated antibodies.

### Expression and purification of recombinant proteins

The human RPA complex was expressed in E.*coli* and purified according to protocol^32^. Briefly, RPA complex was expressed in E. *Coli*. Cells were harvested and resuspended in HI buffer (30 mM HEPES, 0.25 mM EDTA, 0.25% myo-inositol, 1 mM DTT, 0.01% NP40). After sonication, lysates were centrifuged at 14,000 rpm for 10 min. the supernatant was incubated overnight at 4 °C in Affi-Gel Blue matrix (Bio-Rad, Hercules, CA) column. After incubation, beads were washed with HI-80mM KCl and RPA complex were eluted with HI-1.5 mM NaSCN. After elution, lysates are incubated overnight 4 °C in Hydroxylapatite column (Bio-Rad, Hercules, CA). After incubation, column were washed using HI buffer and RPA complex were eluted with HI-80 mM KPO_4_. RPA complex were incubated overnight 4 °C in Mono-Q (Pharmacia) column. After incubation, column were washed using HI- 40 mM KCl, and HI-100mM KCl and RPA complex were eluted with HI-300 mM KCl. GST-tagged ZFP161 (WT, N- terminal, M- terminal, C- terminal) proteins were expressed in E. *coli*. Cells were harvested and resuspended in NETN buffer. After sonication, lysates were centrifuged at 14,000 rpm for 10 min. The supernatant was incubated overnight at 4 °C with Glutathione beads. After washing with NETN buffer, the protein-conjugated beads were used for in vitro pull-down assay or proteins were eluted for in vitro assay.

### DNA templates

To generate the DNA template for in vitro assay, 10 pmol of ssDNA was incubated with 10 pmol biotinylated ssDNA in annealing buffer. The solution was heated at 95 °C for 3 min and cooled at a rate of one degree per minute. Biotin-labeled annealed DNA was incubated with streptavidin bead in binding buffer for 30 min at room temperature. Beads were washed twice with washing buffer and used for further experiments. Sequence of DNA oligonucleotides used to generate the DNA fork substrates for this study; 5′-ATAAATATTTTTTATTAATAATAGATCACCTTTCTTTCTCTTCTCCCCTT-Biotin3′, 5′-TTCCCCTCCTCTCCTTCCTTCCTGATCTATTATTAATAAAAAATATTTAT-3′^33^.

### In vitro binding assay

For GST-ZFP161 in vitro binding assay, ssDNA-beads were incubated initially with purified RPA1/2/3 complex for 30 min in binding buffer (40 mM Tris–HCl, pH 7.5, 150 mM NaCl, 10 mM MgCl_2_, 100 μg ml^−1^ BSA, 1 mM DTT, 1 mM ATP, and 10% glycerol) followed by ZFP161 conjugated GST beads for 30 min. For ssDNA pull-down assay, biotin labeled ssDNA and streptavidin beads were incubated with lysate from cells with or without ZFP161 expression. Beads were rinsed twice with NETN buffer. The product was processed by boiling the sample with 2x Laemmli buffer and performing SDS PAGE^8^.

### Colony formation assay

500 cells were plated in triplicate in each well of 6 well plates. After 16 h, cells were treated with camptothecin (CPT, nM), hydroxyurea (HU, mM), ultra violet (UV, J m^−2^), or Fluorouracil (5-FU, µM) and left for 10–14 days at 37 °C to allow colony formation. Colonies were stained with Giemsa and counted. Results were normalized to plating efficiencies.

### DNA fiber assay

To check fork symmetry and fork speed, cells were first labeled with ldU 25 μM for 20 min, washed twice with media, and labeled with CldU 200 μM for 20 min. Fork symmetry was analyzed by measuring the length of red fiber on each side. Fork speed was analyzed by measuring red fiber length. New firings of origins were measured by counting only red fibers and compared to total fiber numbers. For restart efficiency of stalled replication forks, cells were labeled with ldU 25 μM for 20 min and then washed twice with media. After washing, cells were treated with HU 4 mM for 2 h. After being washed with media, cells were recovered in fresh medium with CldU 200 μM for indicated time point. Cells were then trypsinized and resuspended in PBS to a concentration of 2.5 × 10^5^ cells ml^−1^. Then, cells were diluted 1:40 with unlabeled cells at the same concentration, and 5 μl of cells was mixed with 15 μl of lysis buffer (200 mM Tris-HCl, pH 7.4, 50 mM EDTA and 0.5% SDS) on a clean glass slide. After 8 min incubation, the slides were tilted at 15° to horizontal, allowing the lysate to slowly flow down along the slide. The slides were then air-dried, fixed in 3:1 methanol/acetic acid and stored at 4 °C overnight. The slides were treated with 2.5 M HCl for 1 h, neutralized in 0.1 M Na_2_B_4_O_7_, pH 8.5, and rinsed three times in PBST (PBS with 0.1% Tween-20).The slides were then blocked in blocking buffer (PBST containing 1% BSA)for 20 min and incubated with anti-BrdU antibody (BD Bioscience: 347580, Abcam: ab6326) in blocking buffer at 37 °C for 1 h. After washing, secondary antibodies were diluted in PBS containing 1% BSA and incubated with cell at room temperature for 1 h. After incubation, the slides washed once with low-salt TBST (36 mM Tris–HCl pH8.0, 0.5 M NaCl, 0.5% Tween-20) and 3 times with PBST. After washing, cells were mounted with anti-fade solution and visualized using a Nikon eclipse 80i Fluorescence microscope. All fiber lengths were measured using Image J.

### Generation of ZFP161^−/−^ mouse model

To generate a mouse model lacking the functional ZPF161, ZFP161 deficient embryonic stem (ES) cells (CE0112) were injected in C57BL/6NHsd blastocyst. The ZFP1614 ES cells (CE0112) were purchased from Mutant Mouse Regional Resource Centers (MMRRC). These cells contain nonfunctional ZFP161 protein due to the interruption of ZFP161 gene by a gene trap vector (pGT0LxfTv2). Blastocysts are injected by Transgenic and Knockout Core at Mayo Clinic. All animal work was approved by the Institutional Animal Care and Use Committee (IACUC-A00002875-18).

### Preparation of mouse splenocyte and metaphase spread

Spleen was harvested from mice (6–12 weeks) and ground on 70μm mesh. For quantification of genomic instability, harvested splenocytes were fixed overnight with 70% Ethanol at 4 °C. Fixed cells were permeabilized using 0.25% Triton in PBS with 0.25% Triton-X100 at 4 °C for 10 min. After permeabilization, cells were washed with PBS and stained with γ-H2AX antibody (Millipore) at RT for 2 h. After cells were washed, cells were stained with FITC mouse secondary antibody (Jackson immune Research) at RT for 1 h. After staining, cells were washed and then incubate with PI/RNase solution (Thermo Fisher) at RT for 30 min. The samples were analyzed an Nxt Attune FACS analyzer (Thermo Fisher) and data analyzed with Flow Jo. For metaphase spread, harvested T cells were incubated with concanavalin A (2.5 µg ml^−1^) for 72 h and colcemid (KaryoMAX, GibcoBRL). After incubation, cells were swollen in prewarmed 75 mM KCl at 37 °C for 20 min. After centrifuge, cells were fixed with carnoy’s buffer (3:1 methanol: acetic acid) at RT for 10 min. The cells were spun down for 4 min at 1000 rpm and then supernatant was aspirated. The cells were resuspended with carnoy’s buffer twice. The cells were dropped on slide and dried for at least 10 min. Slides were stained with Giemsa solution (Sigma)^34^. Genomic instability was measured by counting cells that have chromosome breaks and loss.

### Micronucleus assay

Micronucleus assay was performed using 6–12 weeks of age mice. Mice blood samples were mixed with 100 μl PBS supplemented with 1000 U ml^−1^ of heparin (Calbiochem). Mixed blood suspension was then added to 1 ml of methanol and stored overnight at −80 °C. Fixed blood cells were washed with bicarbonate buffer (0.9% NaCl, 5.3 mM NaHCO_3_). The cells were suspended in 100 μl of bicarbonate buffer with 1 μL of FITC-conjugated CD71 antibody (FITC, eBioscience) at 4 °C for 45 min. The cells were washed with bicarbonate buffer and resuspended in PI/RNase solution (Thermo Fisher) at RT for 30 min. The samples were analyzed an Nxt Attune FACS analyzer (Thermo Fisher) and data analyzed with Flow Jo^35^.

### Cell cycle analysis

ZFP161 proficient or deficient cells were treated with 2 mM HU. After 24 h, cells were washed and replaced with fresh media. Cells were harvested at the indicated hours. Cells were fixed in 70% ice-cold ethanol, treated with RNase A, stained with propidium iodide and analyzed on an Attune Nxt Flow cytometry (Thermo Fisher) and data analyzed with Flow Jo.

### Chromatin instability signature

The RNA level of ZFP161 in various tumor types from TCGA, expressed as log (FPKM + 1), was downloaded from UCSC Xena browser. The chromatin instability signatures including the number of telomeric allelic imbalance^36^, the number of large scale transition^37^, homologous recombination deficiency score^38^, the total number of mutations per sample^39^, weighted genomic integrity index^40^, and frequency of loss of heterozygosity^41^ were obtained from Andrea et al. study^42^. The tumors showing lower than the first quartile score, between the first and the third quartile score, or higher than the third quartile score in any chromatin instability feature were defined as low, medium or high with that feature, respectively. The un-paired t-test was used to compare the ZFP161 expression levels.

### Statistics and reproducibility

Data in bar and line graphs are presented as mean ± S.D. of at least three independent experiments. All western blot assays shown here were successfully repeated at least three times.

### Reporting summary

Further information on research design is available in the Nature Research Reporting Summary linked to this article.

## Supplementary information

Supplementary Information

Reporting Summary

## Data Availability

The chromatin instability signatures based on Affymetrix SNP6 genotyping data from the Cancer Genome Atlas are available in the supplementary material of Andrea et al. study (10.1186/s40364-015-0033-4)^42^. The source data for Fig. 6 have been provided as Fig. 6H, I–L panel in Source data file. All data is available from the authors upon reasonable request.

## Source data

Source Data
