# Supplementary material for: ZFP161 regulates replication fork stability and maintenance of genomic stability by recruiting the ATR/ATRIP complex
Source: Nat Commun. 2019 Nov 22;10:5304. doi: 10.1038/s41467-019-13321-z (PMC6876566; doi:10.1038/s41467-019-13321-z)
Supplement: Supplementary file 1 — Supplementary Information [file 41467_2019_13321_MOESM1_ESM.pdf]

Supplementary Information

ZFP161 regulates replication fork stability and maintenance of genomic stability by recruiting the ATR/ATRIP complex

Kim *et al.*

This PDF includes:

Supplementary Figures 1 - 5

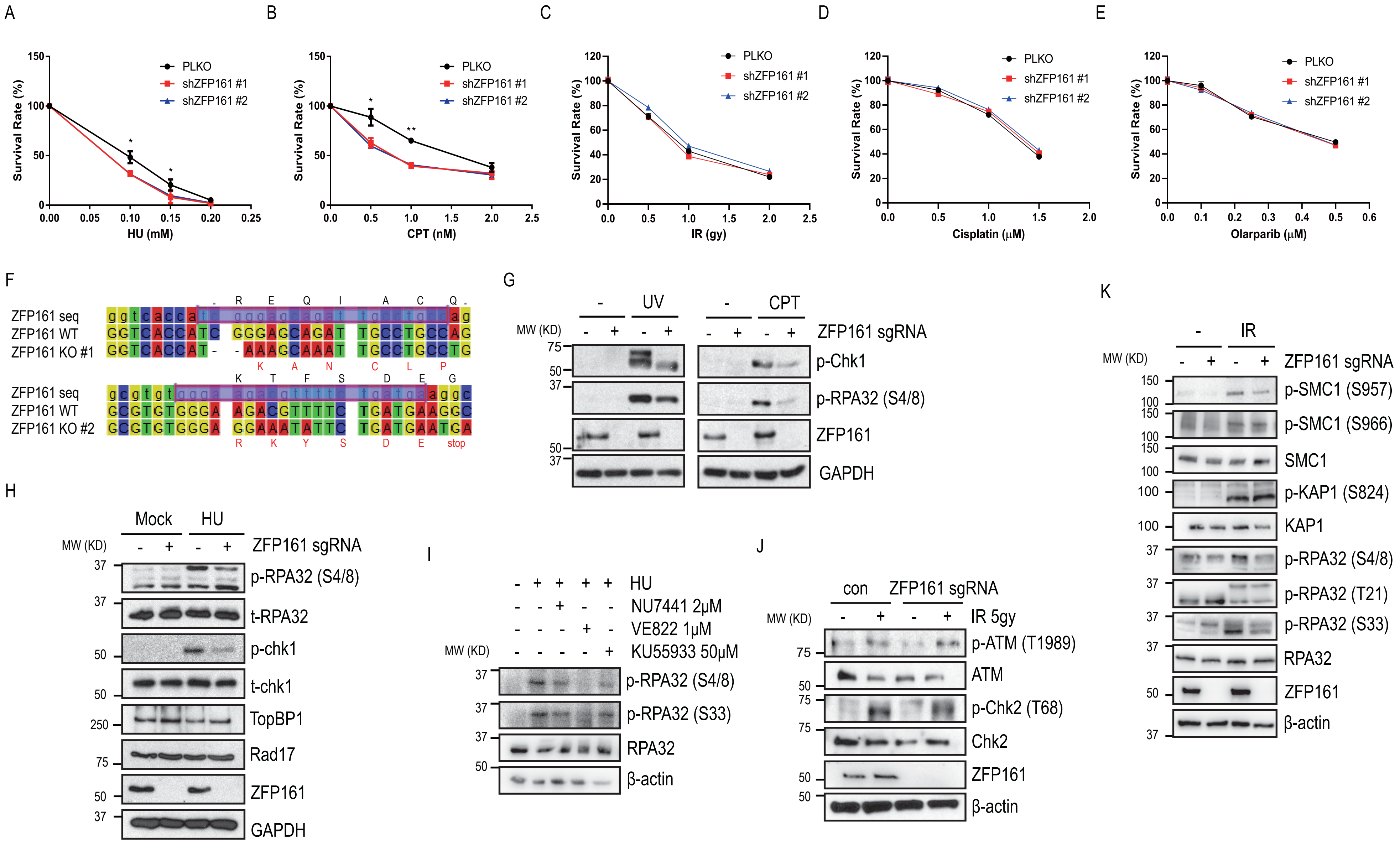

Supplemental Figure 1. ZFP161 is involved in ATR pathway but not ATM pathway

A - E. HCT116 cells with or without ZFP161 expression were plated and treated with Hydroxyurea (HU, mM) (A), Camptothecin (CPT, nM) (B), ionizing irradiation (IR, gy) (C), Cisplatin, μM (D) and Olaparib, μM (E). After 14 days, colony numbers were counted. F. Genomic DNA was isolated and the ZFP161 locus was amplified using PCR. PCR products were analyzed by sequencing. G - H. After 2h UV, CPT (G), HU (H), total cell lysates were immunoblotted using indicated antibodies. I. Cells were pre-treated different inhibitors (VE-822 1μM, NU7441 2μM, KU55933 50 μM) for 1h then cells were treated 10mM HU for 2h. Total cell lysates were immunoblotted using indicated antibodies. J – K. 2h after IR treatment, total cell lysates were blotted using indicated antibodies. The graphs represent mean ± SD, two-tailed, paired t-test; n = 3 independent experiments.

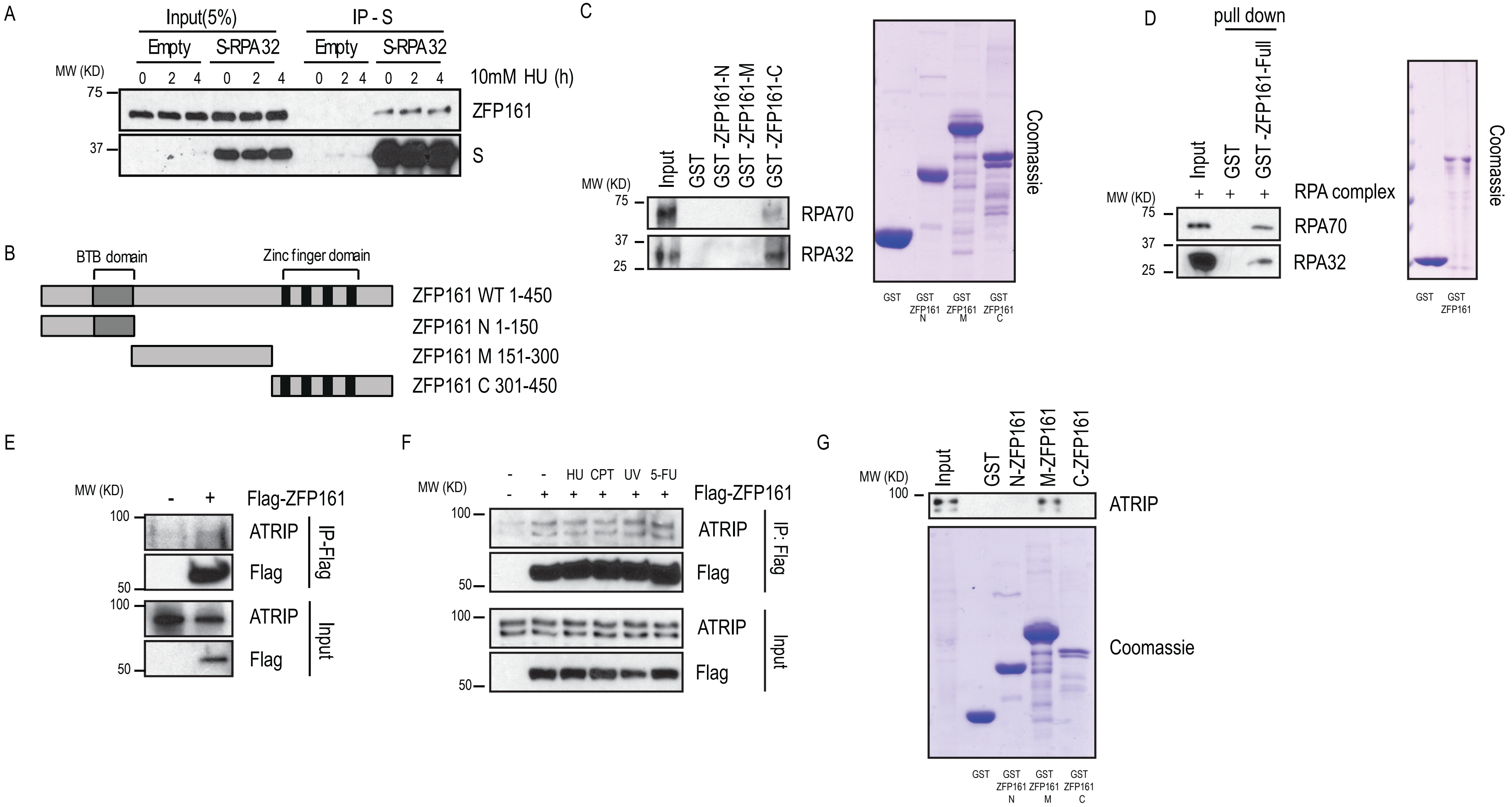

Supplemental Figure 2. ZFP161 directly interacts with the RPA complex and ATR/ATRIP.

A. HEK293T cells were transfected with constructs encoding S-tag RPA32. 48 hours later, cells were incubated with HU for indicated time points. Whole cell lysates were used for immunoprecipitation with S-tagged beads. Blots were probed with the indicated antibodies. B. The schema of ZFP161 constructs used for GST pulldown assay. C. Whole cell extracts were subjected to pulldown using GST-ZFP161 (Full, N-term, M-term, C-term) and immunoblotted with indicated antibodies. D. Purified RPA complexes were incubated with GST or GST-ZFP161 for 30min and immunoblotted with indicated antibodies. E - F. HEK293T cells were transfected with Flag-tagged ZFP161. 48hrs later, cells were treated with the indicated replication stress inducing agents. Whole cell lysates were used for immunoprecipitation with Flag-tagged beads. Blots were probed with indicated antibodies. G. Whole cell extracts were subjected to pulldown using GST-ZFP161 (Full, N-term, M-term, C-term) and immunoblotted with indicated antibodies.

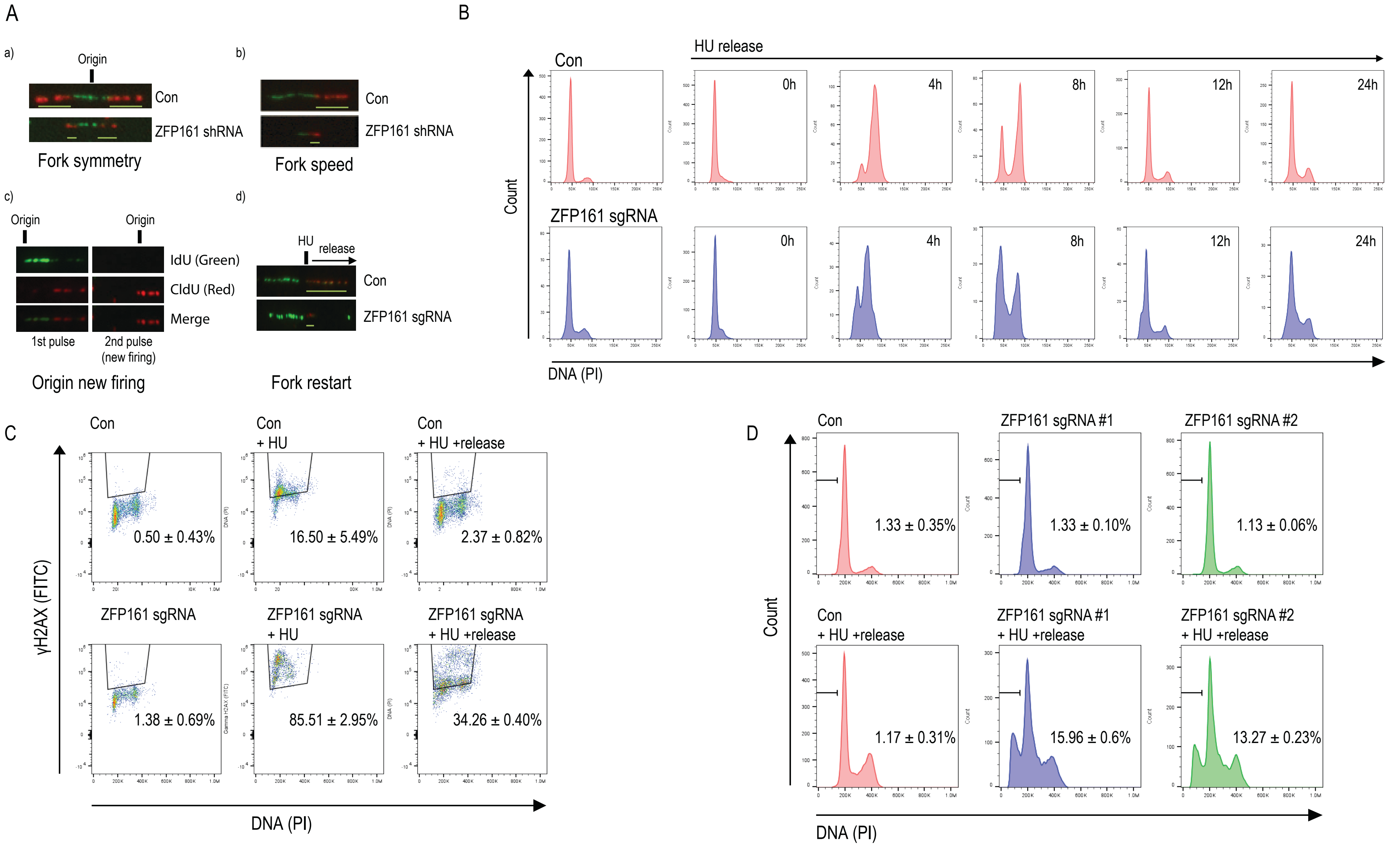

Supplemental Figure 3. ZFP161 enhances stalled replication fork restart

A. Representative images of DNA fibers. Replication parameters observed by DNA fiber assay and their interpretations. B – D. HCT116 cells were treated with 2mM HU for 24 hours and released. Cells were collected and cell cycle profile (D), γ-H2AX (C), and sub G1 population (an indicator of cell death) (D), were determined by flow cytometry. Shown are representative profiles after indicated treatment. The plots represent mean ± SD, two-tailed, paired t-test; n = 3 independent experiments.

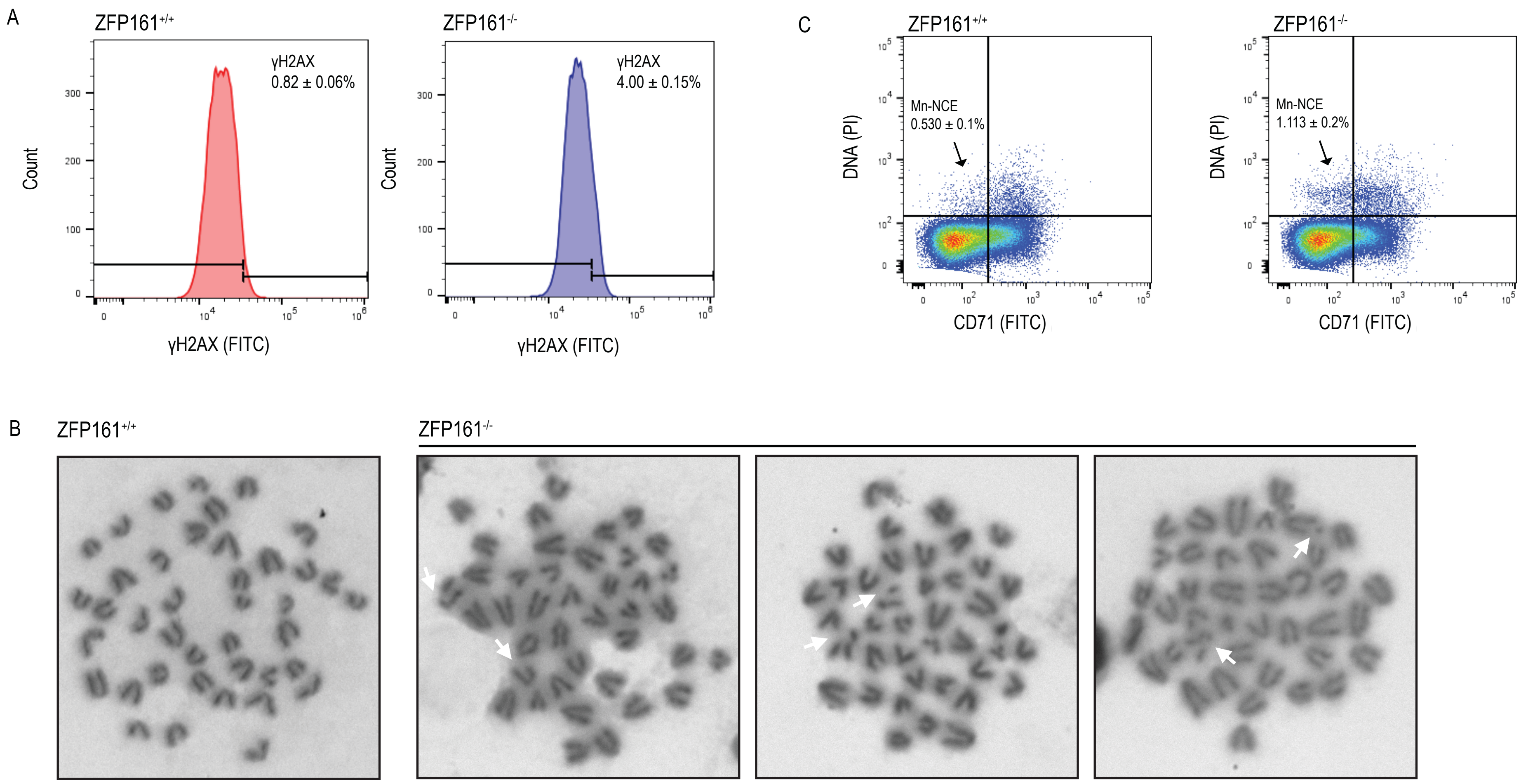

Supplemental Figure 4. ZFP161 Maintains Genomic Stability

A - C. Treatment of mice to assess genomic instability as indicated by γH2AX (A), metaphase spread (B), and population of micronucleated normochromic erythrocytes (Mn-NCE, CD71-PI+) (C). Chromosomal instability was determined by counting cells that have chromosome breaks and loss. White arrows indicate chromosome break or chromosome loss. Data are represent mean ± SD, two-tailed, paired t-test; n = 3 independent experiments. represented as mean ± SD of three independent experiments.

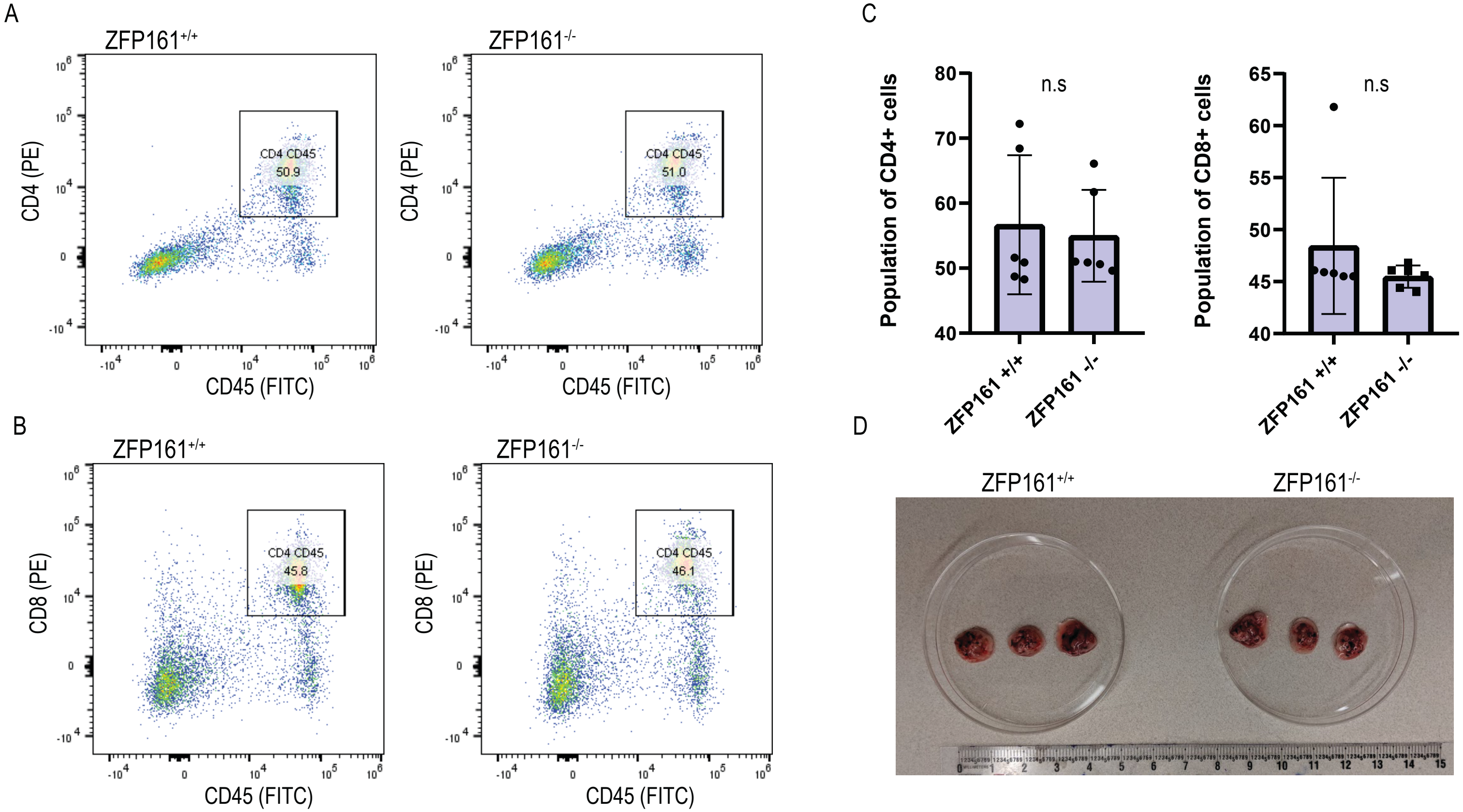

Supplemental Figure 5. ZFP161 does not affect Thymus cellularity

A - C. T cell differentation are determined using indicated antibodies. CD4+, CD45+ (A), CD8+, CD45+ (B) T cells are determined by flow cytometry. The percentage of T cell populations were quantified (C). D. Photograph of thymus isolated from WT (left) and ZFP161 Knockout (right) mice. Data are represent mean ± SD, two-tailed, paired t-test; n = 3 independent experiments.
