# Supplementary material for: ZFP161 regulates replication fork stability and maintenance of genomic stability by recruiting the ATR/ATRIP complex
Source: Nat Commun. 2019 Nov 22;10:5304. doi: 10.1038/s41467-019-13321-z (PMC6876566; doi:10.1038/s41467-019-13321-z)
Supplement: Supplementary file 2 — Reporting Summary [file 41467_2019_13321_MOESM2_ESM.pdf]

nature research

Corresponding author(s): Zhenkun Lou

Last updated by author(s): Oct 10, 2019

Reporting Summary

Nature Research wishes to improve the reproducibility of the work that we publish. This form provides structure for consistency and transparency in reporting. For further information on Nature Research policies, see Authors & Referees and the Editorial Policy Checklist.

Statistics

For all statistical analyses, confirm that the following items are present in the figure legend, table legend, main text, or Methods section.

n/a	Confirmed	
☐	☒	The exact sample size (*n*) for each experimental group/condition, given as a discrete number and unit of measurement
☐	☒	A statement on whether measurements were taken from distinct samples or whether the same sample was measured repeatedly
☐	☒	The statistical test(s) used AND whether they are one- or two-sided<br>*Only common tests should be described solely by name; describe more complex techniques in the Methods section.*
☐	☒	A description of all covariates tested
☐	☒	A description of any assumptions or corrections, such as tests of normality and adjustment for multiple comparisons
☐	☒	A full description of the statistical parameters including central tendency (e.g. means) or other basic estimates (e.g. regression coefficient) AND variation (e.g. standard deviation) or associated estimates of uncertainty (e.g. confidence intervals)
☒	☐	For null hypothesis testing, the test statistic (e.g. *F*, *t*, *r*) with confidence intervals, effect sizes, degrees of freedom and *P* value noted<br>*Give P values as exact values whenever suitable.*
☒	☐	For Bayesian analysis, information on the choice of priors and Markov chain Monte Carlo settings
☒	☐	For hierarchical and complex designs, identification of the appropriate level for tests and full reporting of outcomes
☒	☐	Estimates of effect sizes (e.g. Cohen's *d*, Pearson's *r*), indicating how they were calculated

Our web collection on statistics for biologists contains articles on many of the points above.

Software and code

Policy information about availability of computer code

Data collection	Attune NxT v2.6
Data analysis	GraphPad Prism7, Flowjo v10, Microsoft Excel, R v3.6

For manuscripts utilizing custom algorithms or software that are central to the research but not yet described in published literature, software must be made available to editors/reviewers. We strongly encourage code deposition in a community repository (e.g. GitHub). See the Nature Research guidelines for submitting code & software for further information.

Data

Policy information about availability of data

All manuscripts must include a data availability statement. This statement should provide the following information, where applicable:

- Accession codes, unique identifiers, or web links for publicly available datasets
- A list of figures that have associated raw data
- A description of any restrictions on data availability

Provide your data availability statement here.

Field-specific reporting

Please select the one below that is the best fit for your research. If you are not sure, read the appropriate sections before making your selection.

☒ Life sciences ☐ Behavioural & social sciences ☐ Ecological, evolutionary & environmental sciences

For a reference copy of the document with all sections, see nature.com/documents/nr-reporting-summary-flat.pdf

Life sciences study design

All studies must disclose on these points even when the disclosure is negative.

Sample size	No statistical method was used to predetermine sample size. All experiments were included with multiple biological replicates based on previous experiences.
Data exclusions	No samples were excluded from analysis.
Replication	All results were tested and confirmed with at least three independent experiments.
Randomization	No method of randomization was applied.
Blinding	No blinding assessment was performed as microscopy and FACS data. For manual microscopy, Data were collected randomly choose at least 5 region. For FACs and high-content microscopy data were anlayzed automatically. Exactly the same gate setting was used for all samples.

Reporting for specific materials, systems and methods

We require information from authors about some types of materials, experimental systems and methods used in many studies. Here, indicate whether each material, system or method listed is relevant to your study. If you are not sure if a list item applies to your research, read the appropriate section before selecting a response.

Materials & experimental systems

n/a	Involved in the study	
☐	☒	Antibodies
☐	☒	Eukaryotic cell lines
☒	☐	Palaeontology
☐	☒	Animals and other organisms
☒	☐	Human research participants
☒	☐	Clinical data

Methods

n/a	Involved in the study	
☒	☐	ChIP-seq
☐	☒	Flow cytometry
☒	☐	MRI-based neuroimaging

Antibodies

Antibodies used

The following antibodies were used:
anti-ZFP161 1:1000 (homemade, rabbit)
anti-phospho ATR (T1989) 1:1000 (homemade, rabbit)
anti-phospho RPA32 (S4/8) 1:1000 (homemade, rabbit)
anti-phospho RPA32 (S4/8) 1:1000 (Bethyl Laboratories: A300-245A, rabbit)
anti-RPA70 1:1000 (Bethyl Laboratories: A300-241A, rabbit)
anti-RPA32 1:1000 (Bethyl Laboratories: A300-244A, rabbit)
anti-RPA32 1:1000 (Santa cruz Biotechnology: sc-56770, mouse)
anti-γH2AX 1:1000 (EMD Millipore Crop: 2884537, mouse)
anti-γH2AX 1:1000 (Bethyl Laboratories: A300-081A, rabbit)
anti-PCNA 1:1000 (CST: 2586, mouse)
anti-H3 1:1000 (Proteintech: 17168-1-AP, rabbit)
anti-phospho Chk1 (S345) 1:1000 (CST: 2348, rabbit)
anti-Chk1 1:1000 (Santa cruz Biotechnology: sc-8408, mouse)
anti-phospho Chk2 (T68) 1: 1000 (CST: 2197, rabbit)
anti-Chk2 1:1000 (CST: 6334, rabbit)
anti-phospho ATR 1:1000 (Genetex: GTX128145, rabbit)
anti-ATR 1:1000 (CST: 13934, rabbit)
anti-ATR 1:1000 (Abcam: ab2905, rabbit)
anti-ATR 1:1000 (Novus: nb100-308, mouse)
anti-TopBP1 (Bethyl Laboratories: A300-111A, rabbit)
anti-Rad17 (Santa Cruz: sc-17761, mouse)
anti-ATRIP 1:1000 (CST: 2737, rabbit)
anti-GAPDH 1:5000 (Proteintech: 60004-1-lg, mouse)
anti-β-actin 1:5000 (Sigma: A2228, mouse)
anti-HA 1:2000 (Santa cruz: sc805, rabbit)
anti-HA 1:2000 (Sigma: H6908, mouse)
anti-Flag 1:2000 (Sigma: F1804, mouse)
anti-Flag 1:2000 (Sigma: F7425, rabbit)
anti-Myc 1:2000 (Santa cruz: sc-40, mouse)
anti-Myc 1:2000 (Santa cruz: sc764, rabbit)
anti-GFP 1:1000 (Santa cruz: sc-9996, mouse)
anti-BrdU 1:100 (Abcam: ab6326, rat)

	anti-BrdU 1:1000 (BD: 347580, mouse)<br>anti-RPA32 (Bethyl Laboratories: A300-244A, rabbit, Santa cruz Biotechnology: sc-56770, mouse)<br>anti-phospho RPA32 (S4/8) (Bethyl Laboratories: A300-245A, rabbit)<br>anti-phospho RPA32 (T21) (Abcam: ab109394, rabbit)<br>anti-phospho RPA32 (S33) (Bethyl Laboratories: A300-246A-M, rabbit)<br>anti-CD71 FITC 1:100 (Thermo fisher: 11-0711-82, mouse)<br>anti SMC1 (Epitomics: 2437-1, rabbit)<br>anti-phospho SMC1 (S957) (CST: 4805, mouse)<br>anti-phospho SMC1 (S966) (Bethyl Laboratories: A300-050A, rabbit)<br>anti-KAP1 (Bethyl Laboratories: A300-274A, rabbit)
Validation	Homemade antibodies were validated by knockout and knockdown cells or comparing with other manufactures antibodies. other antibodies were validated by the manufactures.

Eukaryotic cell lines

Policy information about cell lines

Cell line source(s)	HEK293T (ATCC)<br>HCT116 (ATCC)<br>U2OS (ATCC)
Authentication	The Cell lines have been authenticated based on morphological criteria.
Mycoplasma contamination	All cell lines were tested negative for mycoplasma.
Commonly misidentified lines<br>(See ICLAC register)	No commonly misidentified cell lines were used.

Animals and other organisms

Policy information about studies involving animals; ARRIVE guidelines recommended for reporting animal research

Laboratory animals	ZFP161 Knockout mice. All of the mice were on C57BL/6 background. Both adult males and females (~ 8month old) are used in experiments.
Wild animals	No wild animals were used in this study.
Field-collected samples	No filed-collected samples were used in this study.
Ethics oversight	All animal work was approved by the Institutional Animal Care and Use Committee (IACUC) under protocol A00002875-18.

Note that full information on the approval of the study protocol must also be provided in the manuscript.

Flow Cytometry

Plots

Confirm that:

☒ The axis labels state the marker and fluorochrome used (e.g. CD4-FITC).

☒ The axis scales are clearly visible. Include numbers along axes only for bottom left plot of group (a 'group' is an analysis of identical markers).

☒ All plots are contour plots with outliers or pseudocolor plots.

☒ A numerical value for number of cells or percentage (with statistics) is provided.

Methodology

Sample preparation	We used FACS for cell cycle analysis (cell cycle and sub G1) and cell analyze (r-H2AX, Micronucleated NCEs (%), Lin- Sca1+ c-Kit+ cells (%)). Cells were trypsinized and suspended in PBS. Further details of the experimental procedures are provided in the materials and Methods.
Instrument	Attune NxT Flow Cytometer (Thermo fisher SCIENTIFIC)
Software	Attune NxT Flow Cytometer software v2.6
Cell population abundance	N/A

Gating strategy

The FSC/SSC gates defined the single cell population and gated indicated antibodies positive population. For cell cycle, after FSC/SSC gating, PI-A and PI-H were gated for single cell population and analyzed PI-A positive population as cell cycle distribution.

☐ Tick this box to confirm that a figure exemplifying the gating strategy is provided in the Supplementary Information.
